# Mechanistic investigations of diabetic ocular surface diseases

**DOI:** 10.3389/fendo.2022.1079541

**Published:** 2022-12-16

**Authors:** Qingjun Zhou, Lingling Yang, Qun Wang, Ya Li, Chao Wei, Lixin Xie

**Affiliations:** ^1^ State Key Laboratory Cultivation Base, Eye Institute of Shandong First Medical University, Qingdao, China; ^2^ Shandong Provincial Key Laboratory of Ophthalmology, Eye Institute of Shandong First Medical University, Qingdao, China

**Keywords:** diabetic keratopathy, dry eye, neuropathy, epitheliopathy, lacrimal gland, pathogenesis

## Abstract

With the global prevalence of diabetes mellitus over recent decades, more patients suffered from various diabetic complications, including diabetic ocular surface diseases that may seriously affect the quality of life and even vision sight. The major diabetic ocular surface diseases include diabetic keratopathy and dry eye. Diabetic keratopathy is characterized with the delayed corneal epithelial wound healing, reduced corneal nerve density, decreased corneal sensation and feeling of burning or dryness. Diabetic dry eye is manifested as the reduction of tear secretion accompanied with the ocular discomfort. The early clinical symptoms include dry eye and corneal nerve degeneration, suggesting the early diagnosis should be focused on the examination of confocal microscopy and dry eye symptoms. The pathogenesis of diabetic keratopathy involves the accumulation of advanced glycation end-products, impaired neurotrophic innervations and limbal stem cell function, and dysregulated growth factor signaling, and inflammation alterations. Diabetic dry eye may be associated with the abnormal mitochondrial metabolism of lacrimal gland caused by the overactivation of sympathetic nervous system. Considering the important roles of the dense innervations in the homeostatic maintenance of cornea and lacrimal gland, further studies on the neuroepithelial and neuroimmune interactions will reveal the predominant pathogenic mechanisms and develop the targeting intervention strategies of diabetic ocular surface complications.

## Introduction

Diabetes mellitus (DM) is an endemic disease that occurs all over the world, imposing extensive health burden on society (1, 2). Diabetics with prolonged periods of hyperglycemia suffer from numerous complications affecting almost every organ system, including the ocular tissues (3, 4). DM-related ocular complications are the leading cause of blindness, especially in developed countries. Although diabetic retinopathy is the most common and well-known ophthalmic complication, DM also has profound clinically relevant effects on the ocular surface (5, 6).

The corneal tissue composes five stratified layers: the epithelium, Bowman’s layer, stroma, Descemet’s membrane and the endothelium (7, 8). Corneal epithelium is the cornea’s outermost layer, whose integrity is essential to maintaining healthy vision. Corneal stroma, which is populated by keratocytes, represents almost 90% of the thickness of the cornea. Corneal endothelium, a single cell layer between the corneal stroma and anterior chamber, exhibits barrier and ‘pump’ functions to maintain corneal dehydration. In addtition, to maintain a healthy ocular surface, the lacrimal gland and meibomian glands produce tears and lipids to prevent excessive evaporation of the tear film. Dysfunctions of these glands will cause dry eye disease (9, 10).

Although the structure of the ocular surface is relatively uncomplicated, problems with either component may have serious consequences. For DM-related ocular complications, various primary pathological manifestations occur, such as decreased corneal sensitivity, delayed epithelialization after corneal abrasions, basement membrane abnormality, corneal neuropathy, and endothelial decompensation (11, 12). Generally, these changes are referred to as diabetic keratopathy or diabetic neurotrophic keratopathy. Another common diabetic complication in the ocular surface is dry eye, with the involvement of lacrimal functional unit dysfunction (LFUD) (13). These complications drastically influence on the quality of life of patients and are frequently underdiagnosed and underestimated.

Current therapies for DK mainly include topical lubricants, antibiotic ointments, patching, bandage soft contact lenses, and corneal transplantation (14). Nevertheless, these treatments are usually incurable for serious DK, even if in combination. For the treatment of dry eye, identifying effective therapeutics remains an urgent challenge. Thus, research on novel drug targets is vital to the prevention and treatment for diabetic complications on the ocular surface.

Herein, we review recent advances in the pathogenesis of diabetic keratopathy and dry eye. We also evaluated the progress in diagnosis and treatment. These novel findings will shed new light on potential intervention strategies for diabetic ocular surface complications.

## Diabetic keratopathy

Diabetic keratopathy is the most common clinical disease in which diabetes affects the ocular surface. It is a potential vision threatening disease, mainly including epitheliopathy, neuropathy and endotheliopathy.

### Diabetic corneal epitheliopathy

The corneal epithelium consists of 5-7 layers of non-keratinized stratified squamous epithelium, which plays a key role in maintaining corneal transparency and stability. Because the cornea has no blood vessels, and the level of tear glucose level is far less than that of aqueous humor and serum in diabetic patients (15, 16), it is believed that the glucose in corneal epithelial cells is mainly transported from aqueous humor (17). The level of glycosylation in the corneas of diabetic patients increased significantly (18), and the accumulation of glycogen granules was observed in diabetic corneal epithelial cells (19). In diabetic patients, corneal epithelial cells are exposed to persistent high levels of glucose, resulting in various clinical epithelial abnormalities.

Several studies have found that corneal epithelium in diabetic patients tends to have increased fragility, lower cell density, thinner thickness and reduced barrier function (20–22). An electron-microscopic examination of corneal epithelium showed an increased epithelial fragility in specimens of diabetic patients (23). Saini and Khandalavla measured the corneal epithelial fragility of healthy people and diabetic patients using an esthesiometer (20). The results revealed that the average corneal epithelial fragility of diabetic patients was significantly higher than that of healthy people, and that the epithelial fragility of diabetic retinopathy patients increased more significantly. Increased corneal epithelial fragility was also found in Goto Kakizaki rats with type 2 DM (24). A few studies reported that there was no statistical significance in the reduction of corneal basal epithelial cell density in diabetic patients (25, 26). However, more clinical studies have demonstrated that the density of corneal basal epithelial cells was significantly reduced in type 1 and type 2 diabetic patients (21, 27–29), which may be related to the reduction of corneal innervation, impaired of basement membrane and higher turnover rate (21). In the diabetic patients, the mean corneal epithelium thickness was thinner (22, 30) which is associated with the stage of the disease. Similarly, Cai et al. verified the characteristics of the thinning of corneal epithelium and the decreasing density of basal epithelial cells in the rodent model of type 1 diabetes induced by streptozotocin (31). The changes of corneal epithelial density and thickness reflect the imbalance between cell proliferation, differentiation, migration and death. The corneal epithelium has a strong barrier function, making it the first line of defense for the eyeball to resist the external environment. It has long been found that the barrier function of diabetic corneal epithelium is weakened (32–34) which is related to the increase of glycosylated hemoglobin level (34), and correspondingly, diabetic corneas are more prone to infection than healthy people (35–39). *In vitro* studies have proven that high glucose exposure leads to the impairment of the human corneal epithelial cell barrier function, but this change was not caused by the reduced expression of tight junction protein (40).

Clinically, epitheliopathy is characterized by superficial punctate keratitis, recurrent epithelial erosion, persistent epithelial defect and delayed and often incomplete wound healing. In our previous review, according to Semeraro’s classification criteria (41), we summarized the manifestations of mild, moderate and severe diabetic corneal epithelial lesions found in our hospital (4). Corneal abrasions in diabetic patients can cause more serious damage, in some cases leading to basement membrane detachment, and in other cases leading to recurrent corneal erosion (42). Epithelial wound healing is critical for restoring corneal barrier function after injury. Corneal epithelial damage in diabetic patients often takes longer to heal, even does not heal, which is also the main reason why diabetic corneal erosion is difficult to treat (14).

The surgical treatment on diabetic patients will more often lead to subsequent epithelial lesions, such as long-term erosion of epithelial cells and poor healing of epithelial cell defects. It has been confirmed that patients with diabetes who have undergone corneal refractive surgery are at greater risk of developing various epithelial diseases (43–45). Therefore, some ophthalmologists suggest that refractive surgery for diabetic patients should be carefully considered, especially for patients with poor blood glucose control (44–47). A recent study showed that DM is an important risk factor of corneal epithelial defect after vitreoretinal surgery (48). Frequently, diabetic patients with epithelial keratitis after cataract surgery have the characteristics of rapid development, severe epithelial damage, and slow corneal epithelial repair (49). Patients with diabetes are at a greater risk of epithelial debridement due to impaired epithelial wound healing (50).

### Diabetic corneal neuropathy

Corneal nerves, a branch of the ophthalmic division of the trigeminal nerve, enter the peripheral cornea in a radial fashion parallel and then penetrate Bowman’s layer to form the corneal sub-basal nerve plexus, which terminate in free nerve endings in the corneal epithelium and comprises the outermost layer of the cornea and protects cornea from microbial invasion (51, 52). Diabetic peripheral neuropathy (DPN) is the most common complication of diabetes, affecting up to 50% of diabetic patients (53). Recent study reported that the density of corneal nerve fiber and branch, and the corneal nerve fiber length are significantly decreased in diabetic patients (12). Moreover, the loss of 6% or more of corneal nerve fibers per year has been found in 17% of diabetic patients (54, 55). Approximately 39% of diabetic patients experience painful DPN when left untreated (56).

In type 1 and type 2 diabetic patients and animal models, the length, branch and density of corneal nerve fibers in the sub-basal nerve plexus near the corneal epithelium have been found to be reduced, which relates to the severity of diabetic polyneuropathy (24, 31, 57–63). Detailed examination by *in vivo* confocal microscopy has revealed increased corneal nerve tortuosity and thickness in diabetic patients (60, 64–68). Moreover, reduced corneal sensitivity is observed in diabetic patients and animals, and the degree is correlated with the severity of diabetes (60, 63, 67, 69–71). Pritchard et al. reported that corneal sensation threshold was significantly higher for patients with neuropathy compared to those without neuropathy and controls (72). Recent studies have identified corneal sensitivity as a potential marker of diabetic neuropathy (73). In addition, the regeneration of corneal sub-basal nerves is significantly slower in diabetic animals during corneal epithelial wound healing (24, 74). Importantly, the reduction of sub-basal nerve plexus density and corneal sensitivity, which precedes other clinical and electrophysiology tests, could be used as markers for DPN assessment (75, 76). In addition, patients with diabetes often have burning, dryness or painful feeling in the eye (77).

### Pathologic mechanisms

The pathogenesis is difficult to investigate through human epidemiological studies due to too many confounding factors. Therefore, researchers often use animal diabetes models and *in vitro* cell models to study pathogenesis (78, 79). The changes in growth factors, immune cells and signal pathways in diabetic keratopathy have been elaborated in previous reviews (4, 14, 17, 78, 80). Here, we mainly discuss the following aspects.

#### Chronic inflammation

**Figure 1:**
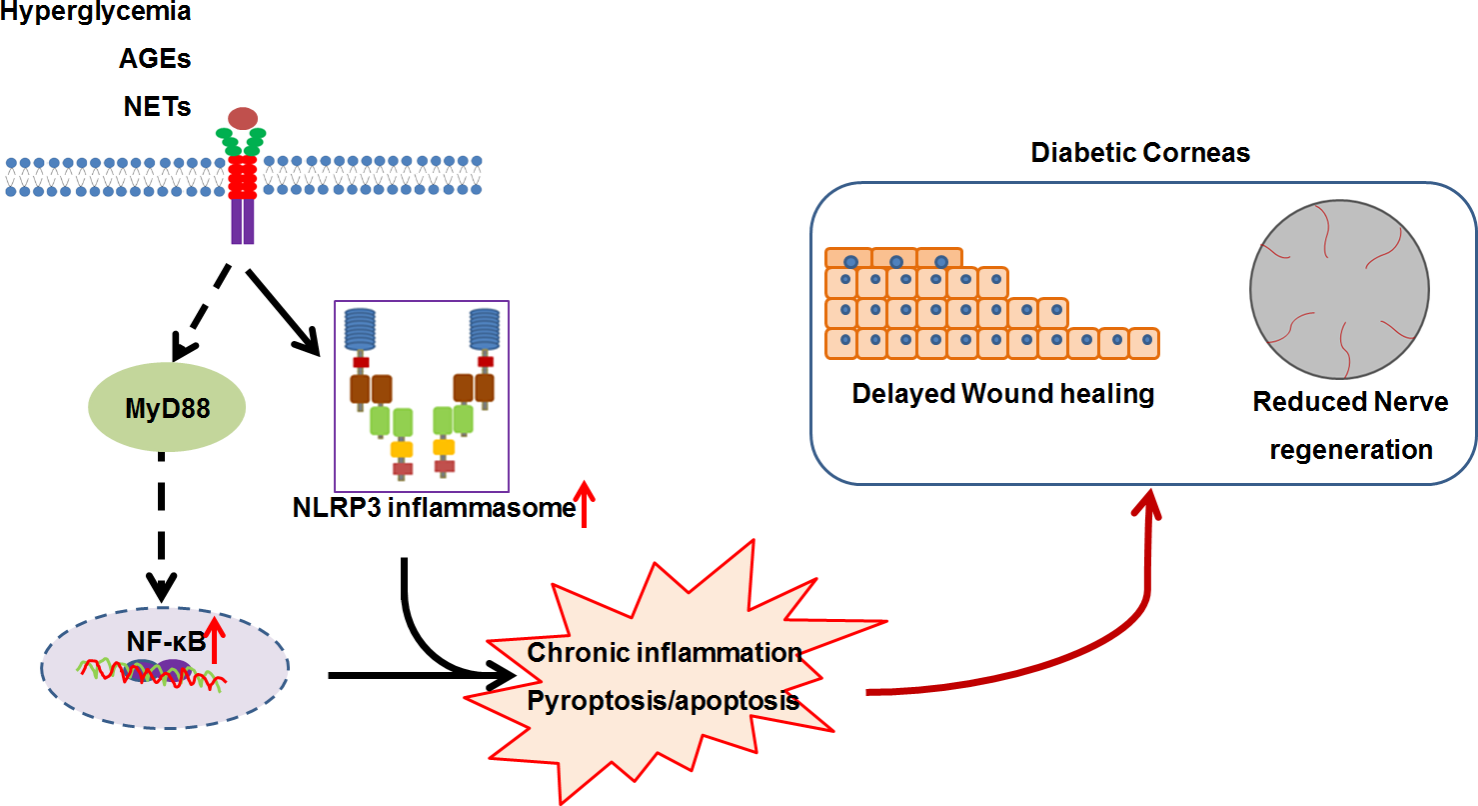
The working model for the low-grade chronic inflammation contributing to diabetic keratopathy. In diabetic mellitus, numerous diabetes-associated danger molecules (such as hyperglycemia, AGEs and NETs), persistently activate NF-κB signaling and NLRP3 inflammasome, resulting in chronic inflammation and pyroptosis, which ultimately postpones corneal epithelial wound healing and impairs re-generation.

As a significant characteristic of DM, low-grade chronic inflammation is regarded as an important mechanism for the development of DM and its complications, including diabetic nephropathy, diabetic retinopathy, and diabetic cardiomyopathy (81, 82). These chronic inflammatory scenarios was triggered and sustained by immune cells and structural cells of specific organs/tissues, which activated innate immunity mainly through pattern recognition receptors (PRRs), such as Toll-like receptors (TLRs) and nucleotide-binding oligomerization domain (NOD)-like receptors (NLRs) (82, 83). Therefore, chronic inflammation theoretically also contributes to the development of DK. Several compelling evidence we found consolidated the pathogenic involvement of chronic inflammation in the development of DK (**Figure 1**).

NOD-like receptor protein 3 (NLRP3) inflammasome, a fully characterized inflammasome, contains NLRP3, adaptor protein ASC, and pro-caspase-1(pro-CASP1), and can be activated by various stimuli, including pathogenic molecules, sterile insults, and metabolic products (84, 85). NLRP3 inflammasome-mediated inflammation plays key roles in the development and progression of DM and its complications, such as diabetic nephropathy (83), diabetic retinopathy (86, 87), and diabetic cardiomyopathy (88). Using genetic and pharmacological approach, we revealed that persistent activation of NLRP3 inflammasome resulted in delayed diabetic corneal wound healing and impaired re-innervation (89, 90). This was supported by the findings of hyper-activation of NLRP3 inflammasome responsible for delayed diabetic skin wound healing and diabetic foot ulcer closure (91–93). Furthermore, we mechanistically revealed that the accumulated advanced glycation end-products (AGEs) promoted hyperactivation of NLRP3 inflammasome through ROS production, ultimately resulting in impaired corneal wound healing and nerve regeneration (89). The findings of AGEs accumulation on the basement membrane of corneal epithelium and Descemet’s membrane in diabetic patients (94–96) were therefore mirrored the possibility of AGEs involving in the DK progression via NLRP3 inflammasome signaling. Generally, the assembly and activation of NLRP3 inflammasome results in the CASP1-dependent secretion of interleukin (IL)-1β and IL-18, as well as gasdermin D (GSDMD)-mediated pyroptosis (97). Yan et al. found that the imbalance of IL-1β and IL-1RA (IL-1 receptor antagonist) in DM corneas inhibited epithelial proliferation and promoted apoptosis, further delaying corneal epithelial healing and re-innervation (98). Inhibition of IL-1β signaling using recombinant IL-1RA and IL-1β neutralizing antibody significantly reversed the postponed diabetic corneal epithelial closure and restored re-innervation (90, 99). In addition to the elevated matured form of IL-1β, the activated form of GSDMD in diabetic corneas after abrasion was also significantly increased (89), which suggested that the GSDMD-executed pyroptosis could be also probably responsible for the excessive inflammation and the impaired corneal would healing and nerve regeneration. Therefore, NLRP3 inflammasome-mediated chronic inflammation is one of important contributors to the pathogenesis of DK, and targeting NLRP3 inflammasome could a promising for DK treatment. Moreover, we also found that blocking TLR4 signaling via TAK-242 expedited diabetic corneal re-epithelialization and nerve regeneration. In addition to receptor of AGEs (RAGE), AGEs also elicit inflammatory response through TLR4 and myeloid differentiation 2 (MD2) (100). AGEs/TLR4 mediated inflammatory response could be another factor attributed to the postponed diabetic corneal wound healing and impaired nerve regeneration.

Under normal conditions, the cornea is endowed with a heterogeneous resident population of antigen-presenting cells, including dendritic cells and macrophages (101–103). Several lines of evidence revealed that specific deletion of dendritic cells or macrophages results in a delayed corneal wound healing in healthy or DM corneas (74, 104–106). Although accumulative evidence indicates the essential role of macrophages and dendritic cells in the pathogenesis and development of DM and its complications (107–110), whether chronic inflammation triggered by macrophages and dendritic cells contributes to DK pathogenesis and progression remains elusive. Fewer neutrophils are usually distributed in normal corneas, but more are recruited after tissue injury or infection. During diabetic corneal wound closure, the number of neutrophils was significantly heightened (111), suggesting a pathogenic role for postponed corneal wound healing and impaired nerve regeneration.

As a component of innate immune system, neutrophils carry out numerous functions, including wound repair (112). During normal wound healing, neutrophils undergo apoptosis after accomplishing their functions, and are subsequently engulfed by macrophages to resolve inflammation (112). However, the DM triggered the neutrophils to NETosis (99, 113). During NETosis, the neutrophils die through releasing web-like chromatin structures loaded with cytotoxic proteins, which is termed as neutrophil extracellular traps (NET) (114). A series of evidence has revealed that NETosis primed by DM resulted in the delayed wound healing and sterile inflammation (99, 113). During diabetic corneal wound healing, NETs production was pronouncedly elevated, and blockade of NETs formation using DNase I or Cl-amidine not only improved inflammation resolution, but also promoted corneal epithelial wound healing and mechanical sensation restoration (115). Besides its crucial role in innate host defense, NETs also fuel inflammatory and autoimmune response, including NLRP3 inflammasome (92, 93, 116, 117). In this regard, NETs would be an essential driver for chronic inflammation during DK pathogenesis and progression.

#### Neurotrophic function

The relationship between corneal nerves and epithelium has been found interdependency and mutual support. The corneal nerves maintain the integrity of corneal epithelium by releasing neurotrophic factors (118). Our laboratory has been committed to studying the role and mechanism of neurotrophic functions in diabetic keratopathy. We found that the levels of many neuropeptides, neurotrophic factors and axon guidance molecules in diabetic corneas were lower than in normal corneas, suggesting that the imbalance of neurotrophic function may be among the critical mechanisms of diabetic keratopathy (4).

**Figure 2:**
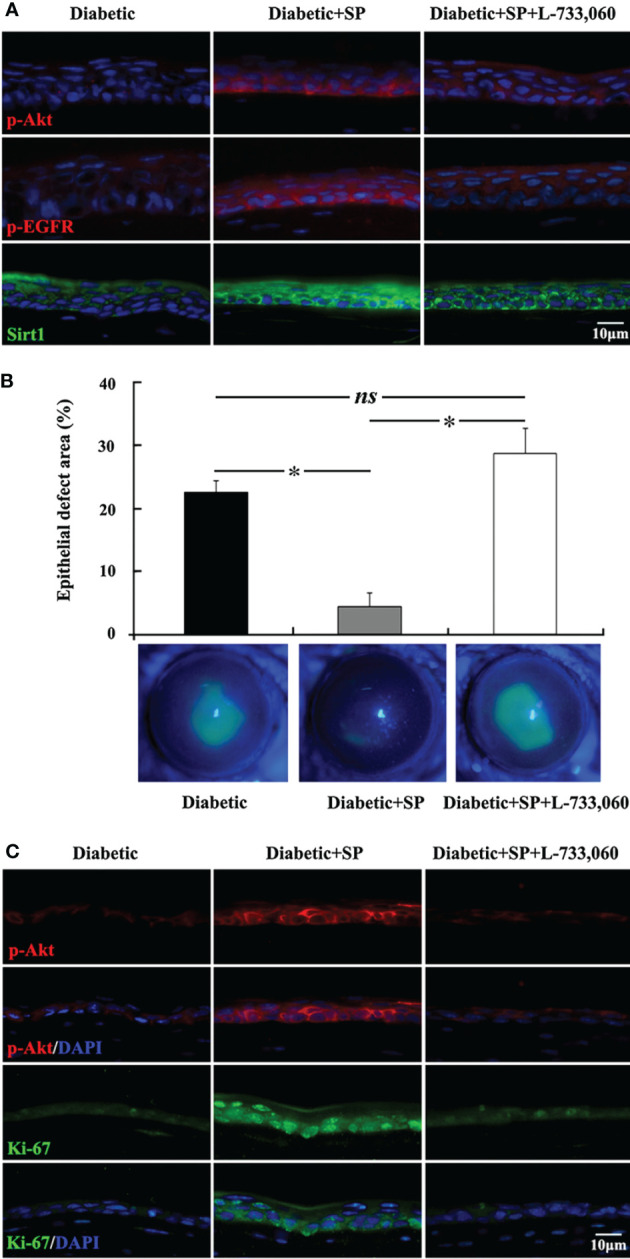
SP-NK-1R signaling regulates diabetic corneal epithelial wound healing. **(A)** In the unwounded corneal epithelium, the elevation of p-Akt, p-EGFR, and Sirt1 level by SP application was attenuated in NK-1 receptor antagonist L-733,060-injected SP-treated diabetic mice. **(B)** L-733,060 injection reversed the promotion of SP on diabetic corneal epithelial wound healing. **(C)** L-733,060 treatment reversed the promotion of SP on p-Akt activation and proliferation in the regenerated corneal epithelium. ns, no significance; *p< 0.05. (ref 126).

Neuropeptides released from the sensory nerve terminals, such as substance P (SP), vasoactive-intestinal peptide (VIP), calcitonin gene-related peptide (CGRP), neuropeptide Y (NPY), and insulin–like growth factor -1 (IGF-1), play important roles in maintenance and nutrition of the corneal epithelium by promoting migration and proliferation (111, 119–127). Substance P (SP) is an 11-amino acid (ARG-PRO-LYS-PRO-GLN-GLN-PHE-PHE-GLY-LEU-MET) neuropeptide expressed in the corneal nerves, cornealepithelium and stromal keratocytes in the cornea (128–131). However, there has been no report on the expression of SP in the resident immune cell population of cornea. We found that SP content in cornea of type 1 diabetic mice decreased significantly. Exogenous SP supplementation markedly promoted epithelial wound healing and cornealsensation recovery by augmenting mitochondrial function, which was blocked by the antagonist of its NK-1R receptor, indicating that SP-NK-1R signaling played a notable role in regulating diabetic epithelial repair (**Figure 2**) (126). Many studies have illustraed that SP-NK-1R pathway can activate multiple signal pathways that promote epithelial growth, migration and adhesion (122, 132–135). Moreover, administration of eye drops containing SP and IGF-1 ameliorated the barrier function by promoting corneal wound healing in rats and rabbits with neurophic keratopathy (121, 136, 137). FGLM, a SP-derivedpeptide (PHE-GLY-LEU-MET), combined with IGF-1, promoted the corneal epithelialwound healing and has been used for the neurotrophic keratopathy treatment in clinical successfully (138, 139). In addition, a tetrapeptide (SSSR) derived from the IGF-1 combination with FGLM also has the synergy in corneal epithelial wound healing (140, 141).

Neurotrophin (NT) is a kind of protein molecule produced by tissues and astrocytes dominated by nerves and necessary for the growth and survival of neurons. Nerves can nourish corneal epithelium, and neurotrophic factors derived from corneal epithelium can also nourish nerves by promoting the growth and survival of nerves. Hyperglycemia attenuates the expression of nerve growth factor (NGF) and glial cell-derived nerve growth factor (GDNF) in the corneal epithelium, while exogenous NGF and GDNF increased the sub-basal nerve fiber density and corneal sensitivity (142). In diabetic mellitus, the content of CNTF and netrin-1 is lessened in diabetic mouse corneas, and we have demonstrated exogenous CNTF improves the corneal epithelial wound healing and nerve regeneration markedly (143). Gao et al. pointed out that dendritic cells are also the main source of CNTF. The reduction of CNTF levels caused by the decrease in dendritic cells during diabetic corneal wound healing is the potential mechanism of diabetic corneal neuropathy (106).

Mesencephalic astrocyte-derived neurotrophic factor (MANF), first discovered as secreted proteins with trophic activity, was expressed in the neuronal and non-neuronal systems especially in high metabolic tissues (144–146). MANF also plays an important role in diabetes. Notably, mice with the konckout of MANF developed diabetes due to increasing apoptotic cell death and reduced proliferation of pancreatic β cells, while recombinant MANF could promote proliferation and prevent cell death (146, 147). In addition, MANF has anti-inflammatory abilities in human pancreatic β cells that protect cells from cell death by repressing the NF-κB signaling pathway (148). MANF has been newly identified in corneas and is reduced in both unwounded and wounded corneal epithelium of diabetic mice. Moreover, recombinant MANF significantly promoted the wound healing of epithelium and nerve regeneration by inhibiting hyperglycemia-induced ER stress and ER-stress related apoptosis (149). Hence, MANF might be a potential therapeutic target for treating diabetic keratopathy.

Besides neuropeptides and neurotrophic factors, there is also a class of factors that play a key role in the repair of nerve innervation, namely axon guidance molecules. These molecules mainly include the Slits family, Netrins family, Ephrins family, Semaphorins family, etc (150). We found that hyperglycemia downregulates netrin-1 expression in corneal epithelium, and the subconjunctival injection of netrin-1 promotes corneal epithelial wound healing and nerve regeneration in diabetic mice. Netrin-1 facilitates the proliferation and migration of corneal epithelial cells under high-glucose conditions. Furthermore, we revealed that netrin-1 inhibited neutrophil infltration, enhanced M2 macrophage transition, and attenuated the expression of pro-infammatory factors in diabetic mouse corneal epithelium *via* adenosine 2B receptor (151). Bettahi et al. revealed that diabetes inhibited the upregulation of Sema3c induced by corneal epithelial injury, but had no effect on Sema3a (152). The diabetic corneal epithelium and nerve regeneration can be promoted by exogenous supplementation with Sema3c (153). The above-mentioned studies suggest that the reduction of axon guidance factors, such as netrin and Sema3c, is partly responsible for diabetic keratopathy.

Some growth factors and metabolites also have neuroprotective effects. The expression of vascular endothelial growth factor (VEGF)-B is decreased in the regenerated diabetic corneal epithelium, and exogenous VEGF-B promotes the regeneration of diabetic corneal nerve fibers by reactivating the PI-3K/Akt-Gsk3ß-mTOR signaling (154). Moreover, VEGF-B also elevates the corneal content of pigment epithelial-derived factor (PEDF). He et al. found PEDF plus docosahexaenoic acid (DHA) could accelerate corneal nerve regeneration in diabetic mice (155). Nicotinamide adenine dinucleotide (NAD) is involved in glycolysis, gluconeogenesis, tricarboxylic acid cycle, and other cellular metabolic reactions, and has essential biological functions (156). Our group demonstrated that NAD^+^ biosynthesis plays an important role in maintaining corneal homeostasis and innervation (157). In diabetic corneas, NAD^+^ content was decreased, and elevated the levels of NAD+ and its precursors NMN and nicotinamide riboside (NR) markedly promoted epithelial and nerve repair by activating SIRT1 and pEGFR, pAKT, and pERK1/2 signaling (158). Another study found that nicotinamide mononucleotide is helpful in improving cell viability and tight junctions in high glucose treated human corneal epithelial cells through the SIRT1/Nrf2/HO-1 pathway (159).

#### Neural ion channels changes

Corneal neurons express a range of membrane channels, including chemical or polymodal nociceptors, mechanonociceptors, and thermal or cold receptors (160, 161). Among corneal afferent neurons, approximately 45% expressed TRPV1, 28% expressed Piezo2, and 8% expressed TRPM8, with 6% of TRPV1 neurons co-expressing TRPM8 (162). The transient receptor potential (TRP) family is thought to transduce environmental and endogenous stimuli to electrophysiological signals. TRPV1 is a well-characterised channel expressed by a subset of peripheral sensory neurons, and canonically mediates inflammatory and neuropathic pain (163). TRPV1 sensitization can be induced by capsaicin. Nowadays, capsaicin 8% patch has been used to alleviate pain in patients with peripheral neuropathic pain, which induced fewer systemic side effects (164–169). Corneal TRPV1 is involved in the maintenance of the corneal structure, re-epithelialization, and inflammation in corneal injury (170). In addition, blinking behavior in guinea pigs related to ocular discomfort is reversed by treatment with the TRPV1 blocker, capsazepine (171). Therefore, corneal TRPV1 may be important for healing corneal tissue, and alleviating the pain in inflammatory disorders of the ocular surface. The depletion of TRPV1+ sensory nerves delayed corneal wound healing by enhancing the recruitment of neutrophils and γδ T cells, increasing the number and TNF-α expression of CCR2+ macrophages and decreasing the number of CCR2– macrophages and IL-10 expression (172). In diabetic conditions, the TRPV1 expression in trigeminal ganglia is increased and the integrity of TRPV1 neurons is important for avoiding alveolar bone resorption and inflammation (173).

Recently, cold receptors have come under greater scrutiny. TRPM8, which is activated by temperatures lower than 25-28°C and menthol, is widely expressed in corneal afferent fibers (174–176). We found that in Trpm8-deficient mice, corneal wound healing is accelerated, while squamous metaplasia occurred in the central corneal opacity after multiple injuries (unpublished data). TRPV1-dependent neuronal sensitization facilitates the release of SP from TRPM8+ cold-sensing neurons to signal nociception in response to cold (177, 178). Overexpression of TRPV1 in TRPM8+ sensory neurons leads to cold allodynia in both corneal and non-corneal tissues without affecting their thermal sensitivity (177). Type 1 diabetic mice exhibit heightened sensitivity to both heat and cold. In diabetic hyperalgesic mice, the thermal hyperalgesia induced by an increase in TRPV1 function is further aggravated by decreased TRPM8 function (179). Abdulhakeem S. Alamri et al. found that the density of corneal nerve fibers in mice fed a high-fat and high-cholesterol diet and those with hyperglycemia had a similar reduction. The reduction of nerve fibers expressing TRPM8 receptors in the corneas of the two models was more significant than that of TrpV1 positive nerve fibers (180).

#### Diabetic autonomic neuropathy

Several influencing factors have been implicated in the pathogenesis of diabetic neuropathy. The hyperglycemic activation of the polyol pathway and protein kinase C may reduce the neuronal blood flow causing direct neuronal damage (181–183). In addition, the increased oxidative stress induced excess nitric oxide production may result in the formation of peroxynitrite and damage to neurons (184, 185). Moreover, the reduction of neurotrophic growth factors, the deficiency of essential fatty acids, and the accumulation of advanced glycosylation end products may also cause less endoneurial blood flow and nerve hypoxia which altered nerve function (183, 186–188). Diabetic neuropathy has been classified as diabetic peripheral and autonomic neuropathies based on pathophysiological characteristics (127). However, few studies have focused on the changes of autonomic nervous system in diabetes keratopathy and its regulatory mechanism.

Diabetic autonomic neuropathy (DAN) is a serious and common complication which has negative impact on the survival and quality of life in patients’ with diabetes (189). DAN may affect many organ systems throughout the body, such as gastrointestinal, genitourinary, and cardiovascular (190). The autonomic nervous system is divided into the sympathetic and the parasympathetic nervous systems. In mammalian corneas, the density of the sympathetic innervations which are from the superior cervical ganglion, vary among species (191). The sympathetic innervations compose about 10–15% of corneal innervations in rabbit, mouse, rat and cat, whereas in primates, they are rarely reported (52, 104, 192). The activation of the sympathetic nervous system has been found in type 1 and type 2 diabetic mice (193–196). In cornea, the activation of sympathetic nervous system may inhibit the wound healing of corneal epithelium and induce the expression of proinflammatory genes in the CD64+CCR2+ macrophages through the β-2 adrenergic receptor (ADRB2) (104). Moreover, we found that the abnormal activation of sympathetic nerve in diabetic mice resulted in the partial depletion of multiple neurotrophins in corneal epithelial cells and dysfunction of limbal stem cells through ADRB2, which further delayed the corneal sensory nerve regeneration and epithelial wound healing (Unpublished data).

The parasympathetic innervations, which are from the ciliary ganglion, exist in different species and vary among in rats, cats, and mice (52, 104, 197). Conversely, the activation of parasympathetic nerves promotes the wound healing of corneal epithelium and enhances the expression of the anti-inflammatory genes in CD64+CCR2- macrophages through α-7 nicotinic acetylcholine receptor (α7nAChR) (104). VIP is secreted predominantly by parasympathetic nervous system. The distinct local macrophages have been found to be activated by VIP, which further modulated inflammation and epithelial renewal. Recently, we found VIP and its receptor are decreased in diabetic corneas in the process of wound healing compared with normal, while exogenous VIP attenuates the wound healing of DM corneas by regulating the wounding inflammatory response and nerve regeneration through Sonic Hedgehog signaling pathway (111).

#### miRNAs and long noncoding RNAs

Generally, miRNA has been proven to be a key regulator of gene expression and can target a variety of molecules that affect cell physiology and disease development. Numerous reports have shown that miRNA relates to the pathology of the diabetic corneal epithelium and nerve damage, making miRNA becoming a promising therapeutic approach for the treatment of diabetic keratopathy.

As the source of corneal nerve fibers, changes in the trigeminal ganglion (TG) caused by diabetes may contribute to corneal neuropathy. Through RNA sequencing, our group found that 68 miRNAs and 114 mRNAs in the TG tissues of diabetic mice diverged from those in normal TG tissues. We predicted that the interaction of miR-350-5p and Mup20, miR-592-5p and Angptl7, and miR-351-5p and Elovl6 may be related to diabetic corneal neuropathy (198). Jianzhang Hu et al. found that inhibiting the expression of miR-181a and miR-34c in TG of diabetic mice promoted the growth of trigeminal sensory neural cells and the regeneration of corneal nerve fibers by regulating autophagic activation (199, 200). Our study revealed that the expression of miR-182 was downregulated in the TG tissue of diabetic mice, which was a key molecule downstream of the endogenous protective gene Sirt1 in TG. And NOX4 was a key target gene for miR-182 to regulate diabetic corneal epithelial and nerve repair (201). Targeting NOX4 and Sirt1 could effectively mitigate the severity of diabetic keratopathy (201, 202).

We also screened differentially expressed miRNAs in the regenerated corneal epithelium of normal and type 1 diabetic mice, and found that miR-223-5p was significantly upregulated, which may be involved in regulating the delay of diabetic corneal wound healing. In the next validation experiment, we confirmed that inhibition of miR-223-5p accelerated the regeneration of diabetic corneal epithelium and nerves, which mediates inflammation response and epithelial cell proliferation through its target gene Hpgds (203). In 2016, our group also found that miR-204-5p, which can directly regulate sirt1, has increased expression in diabetic corneas, and inhibition of miR-204-5p promotes corneal epithelial regeneration by accelerating cell cycle (204).

Compared with normal diabetic mice, diabetic miR-146a KO mice had significantly delayed epithelial wound healing of cornea and skin, and increased neutrophil infiltration. The potential mechanism was that miR-146a KO induced an imbalance in the IL-1β, TNF-α, IRAK1, TRAF6 and NF-κB signaling pathways. Interestingly, there was no difference in corneal wound healing between miR-146a KO and normal mice with normal blood glucose (205). Subsequently, another group’s research in cultured human limbal epithelial cells showed that overexpression of miR-146a reduced the expression of proinflammatory TRAF6, IRAK1 and downstream target NF-κB; and inhibited the expression of cytokine IL-1α, IL-1β, IL-6 and IL-8 and chemokines CXCL1, CXCL2, and CXCL5, which were significantly upregulated in diabetic corneal limbal epithelial cells (206). These studies indicate that miR-146a plays an important role in the regulation of corneal epithelial homeostasis and regeneration under diabetic conditions.

lncRNAs are a class of noncoding RNA molecules with a length of more than 200 nucleotides, which have been reported to play a regulatory role in diabetic complications, retinopathy, pterygium and other eye diseases. Xiaxue Chen and Jianzhang Hu analyzed the differentially expressed lncRNAs (DELs) in the regenerated corneal epithelium of type 1 diabetic and normal corneas. In the diabetic group, 111 upregulated DELs and 117 downregulated DELs were detected. The authors conducted in-depth research on lncRNAs Rik, which is significantly downregulated in diabetes, and found that Rik can be combined with miR-181a-5p as a ceRNA, thus promoting the healing of diabetic corneal epithelial wounds (207).

#### Limbal stem cell dysfunction

The corneal epithelium is self-renewed and regenerated by limbal stem cells (LSCs) that reside in the basal epithelial layer of the limbus, which plays a key role in corneal epithelial wound healing (208–211). A study based on the alteration of LSCs in patients with diabetes found that the expression of markers of LSCs such as ΔNp63α, ATP-binding cassette sub-family G member 2 (ABCG2), N-cadherin, K15, K17, K19, and β1 integrin was decreased significantly in the diabetic limbus (212). *In vitro* cultured LSCs from healthy and diabetic patients were subjected to immunofluorescence staining with LSC markers, and it was also found that the expression of LSCs markers ΔNp63α, PAX6, ABCG2, K15 and K17 in diabetic patients was reduced markedly, especially K15 and K17 (213). Similarly, type 1 and type 2 diabetic mice also showed a significant reduction of LSCs markers in corneal limbus (143, 214). Thus, the loss or dysfunction of the resident LSCs could be responsible for clinically observed delayed corneal epithelial wound healing in diabetic corneas. Therefore, improving the function of diabetic LSCs through genes or growth factors is expected to be an effective means to promote diabetic corneal epithelial wound healing.

**Figure 3:**
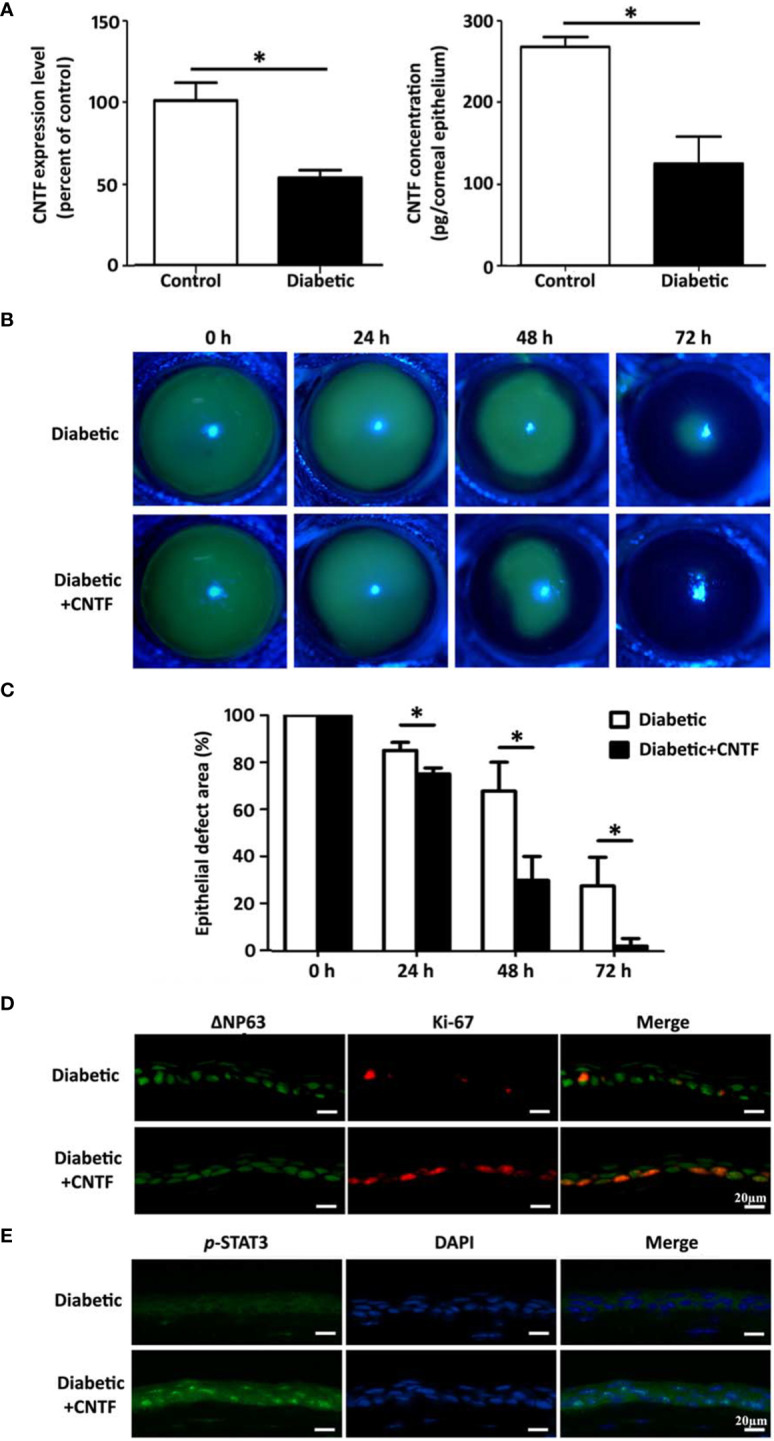
CNTF promotes corneal epithelial wound healing in diabetic mice. **(A)** CNTF is decreased in diabetic corneas both in mRNA and in protein level. **(B, C)** Subconjunctival injection of 50 ng CNTF significantly promotes the corneal epithelial wound healign in diabetic mice. **(D)** The expression of ΔNp63 and Ki-67 in the regenerating corneal epithelium is upregulated after CNTF treatment. **(E)** CNTF activated Stat3 signaling in diabetic wounded corneas. "*p< 0.05. (ref 215).

We found that the expression of neurotrophic factor CNTF was significantly reduced in corneal epithelium of STZ-induced type 1 diabetic mice. Studies in cultured mouse corneal epithelial stem/progenitor cells found that CNTF increases the efficiency of clone formation, promotes cell proliferation, and upregulates the expression level of corneal epithelial stem/progenitor cell-related transcription factors by activating Stat3 signal (143). It can also upregulate MMPs by activating Akt signal to promote the migration of corneal epithelial stem/progenitor cells (215). CNTF supplementation by subconjunctival injection can promote the corneal epithelial would healing both in normal and diabetic mice, and is accompanied by the enhancement of corneal epithelial stem/progenitor cell proliferation activity (**Figure 3**). In contrast, the application of CNTF neutralizing antibody significantly impairs the normal repair function of corneal epithelium. Hiroki Ueno et al. reported that insulin-like growth factor-I (IGF-I) is capable of protecting against corneal stem/progenitor cells and nerve damage in diabetes (214). Taken together, growth factors, such as CNTF and IGF-1, have potential effects in ameliorating limbal stem cell deficiency and treating diabetic keratopathy by enhancing LSCs functions.

Some compounds also have the effect of enhancing the stemness of limbal stem cells, such as ascorbic acid (216), ROCK inhibitor Y-27632 (217), and pluripotin (218). Recently, we found that the proinflammatory cytokines IL-1β and TNF-α were overexpressed during diabetic corneal epithelial wound healing (219). Proinflammatory cytokines can suppress the LSCs markers expression and the colony-forming capacity of corneal epithelial stem cells, as well as destroy the normal ability for corneal epithelial wound healing in a mouse model (220). Proinflammatory cytokines regulate corneal epithelial wound healing through p16Ink4a-STAT3 signaling, and knockdown of p16Ink4a partially restores diabetic corneal epithelial repair defects (221). Yuka Okada et al. confirmed that the sensory nerve TRPV4 is essential for maintaining the stemness of LSCs and is one of the main mechanisms for maintaining corneal epithelial homeostasis (221). Thus, controlling inflammation and maintaining sensory nerve function are beneficial to diabetic corneal epithelial wound healing.

### Diabetic corneal endotheliopathy

#### Clinical manifestation

Corneal endothelial cells (CECs) can be characterized according to the percentage of hexagonal cells (HEX) and the coefficient of variation (CV) (222–225). The previous researches are inconsistent regarding the effect of DM on CEC pleomorphism and polymegathism. Many studies report that the CECs of diabetic patients have a decreased HEX and an increased CV compared to healthy controls (226–230), whereas other studies show no differences (224, 225, 231, 232). Most studies support the hypothesis that DM is associated with worsening CEC pleomorphism and polymegathism. Especially, studies comparing patients with type-1 and type-2 DM (T1DM and T2DM, respectively) found that individuals with T1DM had more remarkable changes in CEC morphology (230, 233, 234).

The rate of cell density loss stabilizes to approximately 0.5% per year (235). Endothelial cell density (ECD) is an indirect marker of endothelial health and function (223–225, 235–239). The rate of CEC loss and the subsequent decrease in ECD speed up in patients with DM (225, 230, 234, 237, 238, 240–244). It should be noted that patients with T1DM (compared to T2DM) and those with a longer disease duration sustain a more severe decline in ECD.

It is widely known that an increase in central corneal thickness (CCT) could serve as one of the earliest signs of CEC dysfunction (245). Many researchers found that T1DM subjects have a higher CCT (238, 245–247). In fact, there have also been reports of a difference in CCT between T1DM and T2DM while few studies have found CCT and DM are unrelated.

#### Pathologic mechanisms

The pathogenesis of corneal endotheliopathy in diabetes is still less studied. The reported mechanisms mainly include mitophagy impairment, endoplasmic reticulum (ER) stress and pyroptosis.

Mitophagy is a highly selective form of autophagy that eliminates dysfunctional or excess mitochondria under stressful conditions, such as hypoxia (248). In our recent study, we demonstrated that hyperglycemia causes abnormal endothelial cell morphology and impaired mitophagy, leading to the accumulation of damaged mitochondria. *In vivo* data also confirmed that increased mitophagy had a protective effect on the CE of diabetic mice. Our results suggest that regulating mitophagy may be a promising strategy for the treatment of diabetic corneal endothelial dysfunction (249).

**Figure 4:**
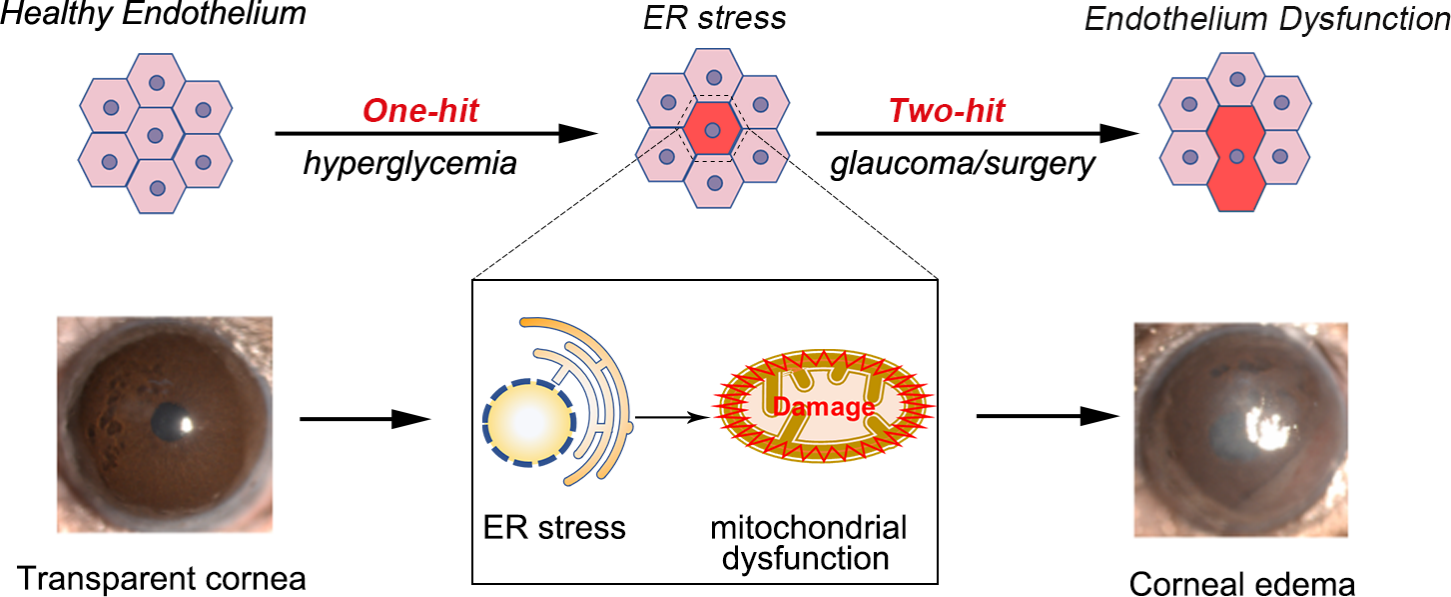
Proposed mechanism of endothelium dysfunction in the diabetic cornea. Endothelial cells from diabetic mice exhibit high levels of ER stress and ER stress appears before morphological changes of the endothelium. Activation of ER stress can promote corneal endothelial dysfunction by triggering mitochondrial dysfunction. Acute injuries, such as glaucoma, can lead to corneal edema and endothelial cell loss.

The ER stress response is a vital regulatory mechanism that maintains intracellular homeostasis (250, 251). The overactivation of the ER stress response and mitochondrial dysfunction are prominent etiological factors in the development of diabetes (252). We observed ER stress response activation in diabetic mice and diabetic human corneal endothelial cells, which induced CEC-specific morphological changes. Persistent ER stress response activation can cause CEC loss and corneal endothelial dysfunction (**Figure 4**). Consequently, in DM, the inhibition of ER stress could mitigate endothelial cell loss and corneal edema *via* the mitochondrial pathway (253).

Pyroptosis is a recently discovered form of programmed cell death that is related to inflammation (254–256). Zhang et al. unraveled the novel role of long non-coding (lnc) RNA KCNQ1OT1 in pyroptosis, whereby KCNQ1OT1-repressed micro-RNA (miR)-214 expression upregulated the expression of the inflammatory molecule Caspase-1 and promoted pyroptosis *in vitro* and *in vivo*. Additionally, KCNQ1OT1 acts as a competing endogenous (ce)RNA that competitively binds miR-214 to regulate Caspase-1 activity, thus promoting diabetic corneal endothelium dysfunction. Further study of the role of KCNQ1OT1 will be critical for understanding the pathogenesis of diabetic corneal endothelium dysfunction and will help identify new biomarkers or potential therapeutic targets to treat this debilitating condition (257).

## Diabetic related dry eye

### Diabetic lacrimal gland disorder

#### Characteristics of diabetic lacrimal gland

Patients with DM may have a higher prevalence of dry eye than the healthy population (258). It has been reported that dry eye disease affects about one-fifth of patients with T2DM and reduces the patients’ quality of life (13, 259, 260). Dry eye may be caused by impaired tear production or excessive tear evaporation and is associated with photophobia, red eyes, vision impairment, local pain, and pruritus. The tear film is the interface between the ocular surface and the environment, and it contains a tightly controlled complement of water, proteins and lipids. LG secretion of proteins and fluid into the tear film is essential for maintaining the health of the ocular surface.

DM impairs tear secretion and induces LG changes. Early studies have identified the involvement of insulin in disorder of LG, such as impaired secretion and a reduction in protein secretion (261, 262). Subsequent studies validated that lipid accumulation in the LG acinar increased with age in a non-obese diabetic (NOD) mouse model. This change is along with lymphocytic infiltration and destruction of the acini. In addition, LG cholesteryl esters obviously increased in these mice (263). Similarly, the polyol pathway was triggered by hyperglycemia in type-2 diabetes, and the accumulation of sorbitol within cells led to cellular edema and dysfunction, which finally resulted in LG dysfunction and decreased tear secretion (264). Recently, He et al. reported that hyperlipidemia affects LG function, including the inhibition of tear secretion, rising lipid accumulation, inflammation, and oxidative stress levels (265). Nakata et al. demonstrated that diabetes suppresses hemodialysis-induced increases in tear fluid secretion, which suggests that the autonomic control of the LG function may be compromised by neuropathy in patients with DM (266). Most recently, our results suggested that streptozotocin-induced type-1 diabetic mice exhibited the early onset of reduced tear secretion and LG weight compared to the symptoms of diabetic keratopathy (267).

#### Pathogenesis of diabetic lacrimal gland

Hyperglycemia, oxidative stress, nerve alterations may play an important role in the development of LG impairment (268) in DM. The detailed mechanisms have become clearer.

**Figure 5:**
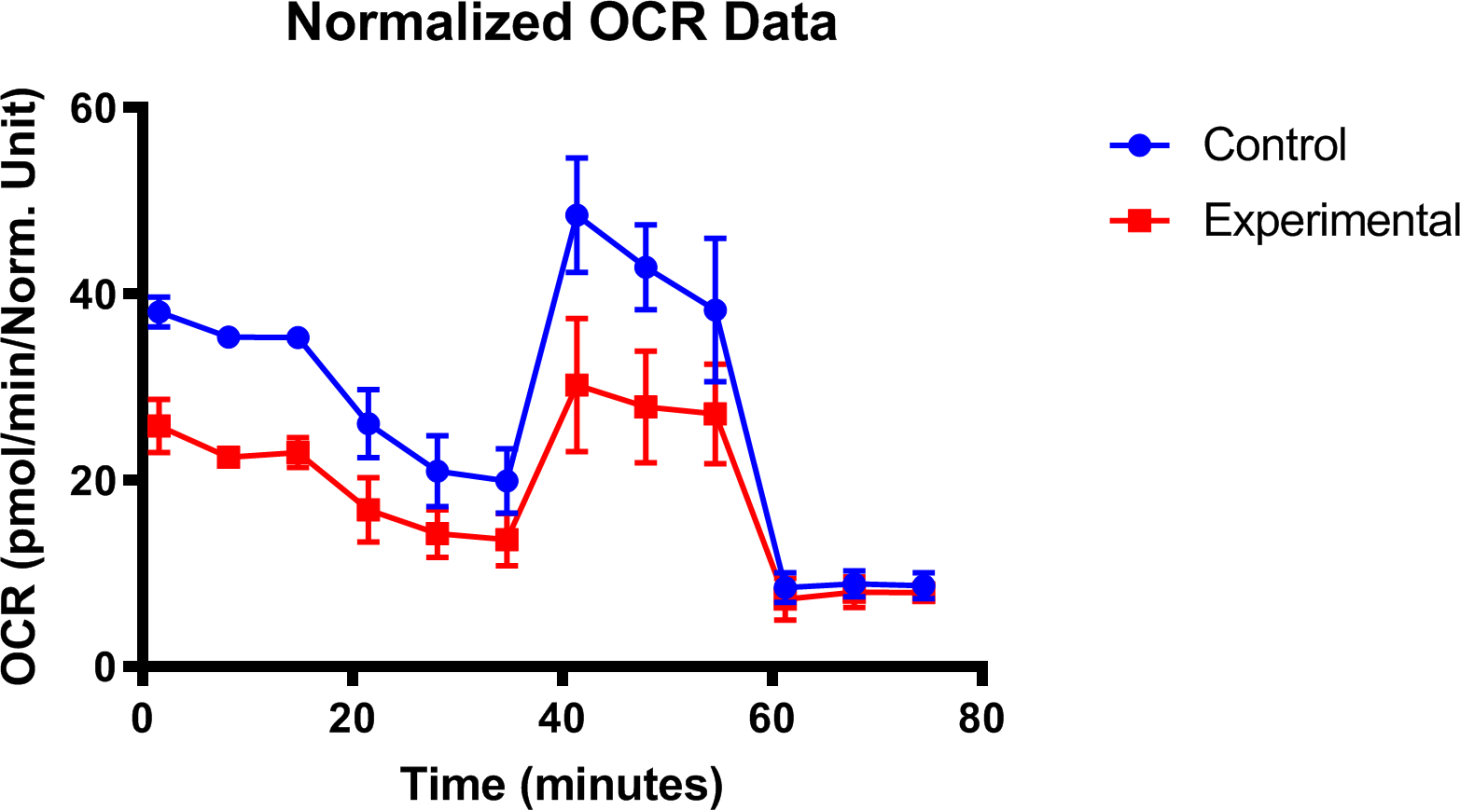
Mitochondrial dysfunction in diabetic lacrimal gland. Lacrimal gland cells from the diabetic mice after 16 weeks were evaluated with the seahorse XFp analyzer (ref. 267).

Mitochondria is the major source of intracellular reactive oxygen species and the target of oxidative damage (269–271). Previous studies had confirmed the existence of oxidative stress and mitochondrial dysfunction in the LG of dry eye mice (272, 273). In the type-1 diabetic model, oxygen consumption rate and basal extracellular acidification rate detection results suggested that the early onset of diabetic dry eye may be due to the susceptibility to a mitochondrial bioenergetic deficit in diabetic LG (**Figure 5**), while the application of mitochondria-targeted antioxidant SKQ1 may ameliorate diabetic dry eye and keratopathy.

It is recognized that inflammation plays a prominent role in the development and propagation of dry eye. Hyperglycemia initiates an inflammatory cascade that generates the innate, adaptive immune responses of the lacrimal functional unit (LFU). The downstream immune-inflammatory regulators have been identified as matrix metalloproteinase-9 (MMP-9), immature antigen-presenting cells (APCs), CD4^+^ helper T cells (TH) subtype 1, and TH17 cell subsets, interferon-γ (IFN-γ) chemokines, chemokine receptors, cell adhesion molecules (CAMs), and interleukin-17 (IL-17) (274).

The neural response that regulates LG fluid secretion is an integral part of the LFU, which consists of the sensory afferent nerves of the cornea and conjunctiva, the efferent parasympathetic and sympathetic nerves that innervate the LG, the LG secretory cells, and the LG excretory ducts (275). Both anatomically and functionally, the parasympathetic system predominates, with overwhelming evidence indicating that the loss of parasympathetic innervation blocks LG functioning (276–283). Research has demonstrated that different densities of sympathetic innervation in glandular areas and the sympathetic denervation of the rabbit LG by ablating the superior cervical ganglion did not alter the LG acinar morphology and induced the denervation supersensitivity of protein secretion (284, 285). In addition, researchers have reported that the electrostimulation of the superior cervical ganglion increased tear secretion (286). However, the involvement of sympathetic stimulation in LG in DM remains poorly understood. Recently, we illustrated that the sympathetic pathway is activated in the pathogenesis of diabetic dry eye and may provide a potential strategy to counteract diabetic dry eye by interfering with sympathetic activity (Unpublished data).

### Diabetic meibomian gland dysfunction

#### Clinical manifestation

Meibomian gland dysfunction (MGD) is an important cause of dry eye, and diabetes may be a risk factor. Studies reveal a high incidence of MGD in patients with diabetes (287–289), especially long-lasting diabetes (290, 291).Yu et al. (287) observed 132 eyes to assess the changes of Meibomian Glands (MGs) in type 2 DM. As the diabetes progressed, they found more MGs dropouts and absence of MGs in the DM group, and MG bubbles density were decreased with shape alterations, such as atrophy, fibrosis, expansion. The opening of glandular duct appeared to be atrophic and cornified. Additionally, lipid layer thickness (LLT), lid margin abnormalities, and tear breakup time (BUT) were significantly changed in diabetic patients; interestingly, the results of LLT were varied in different investigations (289, 290), which deserves further research. More importantly, some studies suggested that diabetes was associated with asymptomatic MGD, and it may be an early sign of ocular discomfort in T2D (290, 292). These findings suggest a lack of association between signs and symptoms. Therefore, it is alert to notice the signs of MGD in the absence of symptoms and perhaps the necessary treatment should be taken to prevent the progression of complications.

While MGD in type 2 DM has been widely investigated in the literature, studies on type 1 DM were very limited. Previous studies reported that BUT were lower in the Type 1 DM group and significantly associated with the duration of DM (293, 294). Semer et al. (295) evaluated the changes of MGs with Type 1 DM and found that in diabetic children, a higher secretion score and total eyelid score appeared. The thinning and shortening of MGs and presence of ghost areas were more common. In Type 1 DM animal model established by streptozotocin (STZ), more signs were founded, such as acini dropout, condensed lipid deposition at the orifice of the MG, disorganized acini and ducts, lipid metabolism disorder compared to those of non-diabetic controls (296, 297). Previous studies have documented peroxisome proliferator activator receptor-γ (PPARγ) plays a dominant role in regulating meibocyte differentiation and lipid synthesis (298, 299). Recent study has confirmed the reduced PPARγ in diabetic MGs, and upregulation of PPARγ could improve the production of lipid (300). Taken together, these indicated that pathological process of MGD could be observed in diabetic model induced by STZ, so, it may be used as vital tool for studying the physiopathology of MGD resulting from hyperglycemia. Generally, the pathogenesis of Type 1 DM differs from that of Type 2 DM, and distinctions in the presentation and progression of MGs between DM types has been seldom reported. Hence, future comparative investigations are necessary.

#### Pathogenesis of diabetic meibomian gland dysfunction

Unlike other sebaceous glands, the lipid secretion of MG is controlled by various neurotransmitter-neuromodulator mechanisms, and disparate neuropeptides/neurotransmitters play a role in the functioning of MG cells (301–303). The continuous proliferation and differentiation of MG cells are the basis for maintaining the secretion of lipids. Neuropathy, one of the most common complications of DM, may lead to MG dysfunction by disrupting the function of MG cells. Peripheral neuropathy may also alter meibum delivery to the ocular surface. Clinical studies have revealed that peripheral neuropathy causes a decline in nerve impulses emanating from the brain and corneal hypoesthesia, which leads to reduced blink rates (69, 70, 304). During blink movement, the muscle could produce a compression force to the tarsal plate and facilitate the delivery of the lipid from the MGs. Therefore, it is speculated that neuropathy leads to a decline in the blinking rate and meibum delivery forces, and ultimately leading to greater MGD prevalence in diabetes patients.

In diabetic patients, laser scanning confocal microscopy (LSCM) displayed the infiltration of inflammatory cells in the interstitial of gland bubble (305). In STZ-induced diabetic mouse model, more CD45 positive cells, such as macrophage and neutrophils, accumulated in MGs (297). Similarly, Yuli at al. found more inflammatory cells and overexpressed inflammatory factors in MG of diabetic rat. Genomic analysis techniques revealed that inflammation-related genes were upregulated in type 2 diabetic mice (306). In addition, more studies have demonstrated that the lipid homeostasis is related to the inflammation (307, 308). Many lipid species could regulate inflammatory responses. In turn, inflammation can alter the lipid metabolism. As a systemic metabolic disease, DM is closely associated with the lipid metabolism, and it has been recognized that diabetes induces the disruption of lipid homeostasis in MGs (297, 309). It was suggested that phospholipids (PLs) may play a key role in the inflammatory reaction. A higher level of PLs was observed in the meibum with DM, and the overexpression of PLs could release more inflammatory mediators, such as free fatty acid (FFA). FFA was considered to be toxic hydrolysate generated by microbial lipases from normal lipids, which would conversely induce inflammation and hyperkeratinization, thus damaging the ocular surface and MGs (310).

## Potential treatment options

Diabetic ocular surface diseases is treated by local symptomatic treatment (such as the use of steroids to treat epithelial defect) on the premise of systemic control of blood glucose (such as insulin injection). However, the existing primary treatment methods cannot fully meet the treatment needs of diabetic ocular surface diseases, so it is necessary to find alternative treatment targets.

Stem cells therapy have been proposed as an emerging treatment option for diabetic keratopathy. Mesenchymal stem cells (MSCs) are a good choice for stem cell therapy due to their pluripotency and regenerative potential (311–314). MSCs exist in various tissues, including bone marrow, peripheral blood, adipose tissue, placenta, nervous tissue and so on. MSCs are known to play an important role in regulating tissue repair and immune inflammation through direct or indirect mechanisms. Our study based on bone marrow mesenchymal stem cells (BM-MSCs) on diabetic corneal wound healing found that the local transplantation of BM-MSCs significantly promoted the repair of corneal epithelium in type 1 diabetic mice. In mechanism, BM-MSCs alleviate diabetic corneal impairment by promoting the activation of corneal epithelial stem/progenitor cells and accelerating the polarization of macrophages to anti-inflammatory M2 phenotypes by secreting tumor necrosis factor-a–stimulated gene/protein-6 (TSG-6) (315).

Based on its ability to self-renew and promote regeneration, hemopoeitic stem cell (HSC) is another potential adult stem cell for disease therapy. Maha et al. assessed the possible effect of HSC therapy on STZ-induced diabetic keratopathy in albino rat and found that a tail vein injection of HSC ameliorated the changes of cornea and conjunctival epithelium caused by diabetic keratopathy (316).

Many studies in stem cell therapy have been conducted to restore corneal functioning, including autologous/allogeneic limbal stem cell transplantation (317), embryonic stem cells (ES)/induced pluripotent stem cells (iPS)-induced corneal cells (318, 319) and various adult stem cell (320, 321) treatments. Some have entered clinical trials; however, stem cell therapy in the field of diabetic keratopathy is still in its early stages. Although MSC and HSC transplantations have certain application prospects at the animal level, they are still far from clinical application, and further exploration is needed in the future.

Considering that cornea, lacrimal gland and meibomian gland are densely innervated, and neuropathy is one of the most common, complex and serious complications of diabetes patients, treatment based on neural regulation has also been emphasized. Exogenous supplementation of sensory neuropeptide SP, CGRP and parasympathetic neuropeptide VIP has been proven to effectively promote the regeneration of corneal epithelium and nerves in the experimental stage. As mentioned above, the therapeutic effects of various neurotrophic factors and axon guidance molecules on diabetic ocular surface diseases have also been successively verified in diabetic animal models. It is worth mentioning that Cenegermin (Oxervate™), an ophthalmic eye drops mainly composed of recombinant human NGF, was recently approved by the FDA for the treatment of neurotrophic keratopathy (322). In addition, our latest study found that sympathetic overactivation caused by diabetes also participated in the pathogenesis of diabetes keratopathy and diabetes related dry eye (Unpublished data). Sympathetic nerve-targeting regulation may also be a potential therapeutic target for diabetic ocular surface disease.

In addition, recent research has also revealed many other new methods to treat corneal epithelial defects, including the application of natural Chinese medicine (such as lycium barbarum polysaccharide) (323), various cell derived exosomes (324), and biological materials (such as hydrogel) (325). The mechanism revealed by these studies has something in common with the pathogenesis of DK, and maybe also used for developing new DK treatment methods, which may eventually open up a new way for developing new treatment methods to improve corneal wound healing.

## Conclusion

With increasing clinical evidences of ocular surface damage in diabetic patients, ophthalmologists have gradually recognized the harm of diabetic ocular surface complications, and more basic ophthalmic research has focused on the disclosure of the pathogenesis and potential therapeutic targets of diabetic ocular surface complications.

The defined pathogenesis of diabetes keratopathy includes the accumulation of advanced glycation end products, the imbalance of growth factors and signaling pathways, the occurrence of persistent inflammation, the decline of neurotrophic function, the dysfunction of stem cells, the impairment of mitochondrial function, excessive oxidative stress, etc. Therefore, controlling inflammation and excessive oxidative stress, improving the function of stem cells and mitochondria, and targeting relevant growth factors, neurotrophic factors and signal pathways will be the direction of developing new targets for DK treatment, and guiding the clinical treatment of DK.

Diabetic dry eye was found to be closely associated with the abnormal mitochondrial function of lacrimal gland and the abnormal lipid metabolism of meibomian gland. For the treatment of dry eyes in diabetes, attention should be paid to improving tear secretion and meibomian gland lipid metabolism. Animal experiments have confirmed that promoting mitochondrial function has a good therapeutic effect on diabetic dry eyes, providing a basis for future clinical applications.

Clinical prospective studies have discerned that the early clinical symptoms of diabetic ocular surface complication are dry eye and corneal nerve degeneration, suggesting that early diagnosis should first examine corneal nerves changes using confocal microscopy and examine dry eye related clinical indicators. Further study on the interaction between neuro-epithelium and neuro-immunity will help to reveal the key pathogenic mechanism and formulate targeted intervention strategies for ocular surface complications of diabetes.

## Author contributions

LX and QZ contributed to the manuscript design, discussion and revision. LY, QW, YL and CW contributed to the manuscript preparation and writing. All authors contributed to the article and approved the submitted version.
